# Drought response of water-conserving and non-conserving spring barley cultivars

**DOI:** 10.3389/fpls.2023.1247853

**Published:** 2023-10-24

**Authors:** Mercy Appiah, Issaka Abdulai, Alan H. Schulman, Menachem Moshelion, Elvira S. Dewi, Agata Daszkowska-Golec, Gennady Bracho-Mujica, Reimund P. Rötter

**Affiliations:** ^1^ Department of Crop Sciences, Tropical Plant Production and Agricultural Systems Modelling (TROPAGS), University of Göttingen, Göttingen, Germany; ^2^ Production Systems, Natural Resources Institute Finland (LUKE), Helsinki, Finland; ^3^ Institute of Biotechnology and Viikki Plant Science Centre, University of Helsinki, Helsinki, Finland; ^4^ Institute of Plant Sciences and Genetics in Agriculture, The Robert H. Smith Faculty of Agriculture, Food and Environment, The Hebrew University of Jerusalem, Rehovot, Israel; ^5^ Department of Agroecotechnology, Faculty of Agriculture, Universitas Malikussaleh, Aceh Utara, Indonesia; ^6^ Institute of Biology, Biotechnology and Environmental Protection, University of Silesia in Katowice, Katowice, Poland; ^7^ Centre for Biodiversity and Sustainable Land Use (CBL), University of Göttingen, Göttingen, Germany

**Keywords:** drought ideotype, drought resilience, intermediate drought, conserving and non-conserving water-use behavior, spring barley, water-use efficiency, yield stability

## Abstract

**Introduction:**

Breeding barley cultivars adapted to drought requires in-depth knowledge on physiological drought responses.

**Methods:**

We used a high-throughput functional phenotyping platform to examine the response of four high-yielding European spring barley cultivars to a standardized drought treatment imposed around flowering.

**Results:**

Cv. Chanell showed a non-conserving water-use behavior with high transpiration and maximum productivity under well-watered conditions but rapid transpiration decrease under drought. The poor recovery upon re-irrigation translated to large yield losses. Cv. Baronesse showed the most water-conserving behavior, with the lowest pre-drought transpiration and the most gradual transpiration reduction under drought. Its good recovery (resilience) prevented large yield losses. Cv. Formula was less conserving than cv. Baronesse and produced low yet stable yields. Cv. RGT’s dynamic water use with high transpiration under ample water supply and moderate transpiration decrease under drought combined with high resilience secured the highest and most stable yields.

**Discussion:**

Such a dynamic water-use behavior combined with higher drought resilience and favorable root traits could potentially create an ideotype for intermediate drought. Prospective studies will examine these results in field experiments and will use the newly gained understanding on water use in barley to improve process descriptions in crop simulation models to support crop model–aided ideotype design.

## 1 Introduction

Drought, as one of the most detrimental climate hazards, is projected to occur more frequently and increase in severity by the end of the 21st century (Dai et al., 2018; Spinoni et al., 2018; Grillakis, 2019; IPCC, 2023). The production of barley, an important cereal for animal feed, malting, and human consumption (Yawson et al., 2017; Hoyle et al., 2020; Yawson et al., 2020), has already been affected by drought-induced yield penalties. For example, over 1964–2015, yield losses amounted to about 9% in Europe (Brás et al., 2021). In 2018, a year marked by climate anomalies of historical dimension, drought coinciding with exceptionally high temperatures caused notable yield reductions in various regions of Europe, resulting in a sharp price increase (extra 60 € per ton) for barley (Toreti et al., 2019; Beillouin et al., 2020).

Drought can interfere with cereal growth, biomass, and yield production by various mechanisms (e.g., Chaves et al., 2003; Barnabás et al., 2008; Aroca, 2012; Lipiec et al., 2013; Kadam et al., 2014; Fahad et al., 2017). The extent of plant damage depends on drought severity and duration as well as on the growth stage, whereby the reproductive stages are the most vulnerable (Wells and Dubetz, 1966; Kadam et al., 2014; Fahad et al., 2017). Among the various plant drought response mechanisms to maintain adequate hydration are drought avoidance, e.g., by flowering and setting seed before drought, increasing water uptake through deep roots, or limiting plant water loss under drought through stomata closure (Chaves et al., 2003; Farooq et al., 2009; Vilagrosa et al., 2012; Shavrukov et al., 2017).

Stomatal aperture and closure are regulated by guard cell turgor pressure (Bertolino et al., 2019), which, in turn, is controlled by a complex regulatory network (Kollist et al., 2019). The evaporative demand of the air, expressed as the vapor pressure deficit (VPD), is the main force driving water movement from the roots to the leaves. Evaporative demand under reduced water supply from the soil can directly decrease guard cell turgor pressure or trigger the response pathway, leading to stomatal closure. Stomata closure buffers the increase in xylem tension, which develops as plant water potential decreases due to limited water supply from the roots. If xylem tension continues to increase, then the resulting xylem cavitation–induced embolism would damage the plant hydraulic system and cells and induce wilting due to a loss of turgor pressure (Jones and Sutherland, 1991; Vilagrosa et al., 2012; Johnson et al., 2018; Johnson et al., 2020).

Depending on the timing of stomata closure, plant behavior can be characterized along a continuum of responses to soil water content (SWC). Production-maximizing plants show high stomatal conductance (*gs*) and transpiration under well-watered conditions and only close stomata at low SWC. Under drought, these water–non-conserving plants (also referred to as “anisohydric”) maintain a high transpiration rate and thereby allow the leaf water potential to drop to comparatively low levels (Domec and Johnson, 2012; Sade et al., 2012; Gallé et al., 2013; Moshelion et al., 2015). Anisohydric behavior allows xylem tension and cavitation risk to increase to a certain degree. As soil moisture depletion continues, xylem tension increases further until a threshold level is reached, at which the plant must close its stomata if it is to avoid catastrophic xylem failure (Jones and Sutherland, 1991; Johnson et al., 2018; Bertolino et al., 2019). In contrast, water-conserving plants (also referred to as isohydric; Sade et al., 2012; Moshelion et al., 2015) limit *gs* early after the onset of drought (i.e., at comparatively higher SWC) and can maintain a constant leaf water potential (Domec and Johnson, 2012; Gallé et al., 2013; Moshelion et al., 2015). As soil moisture depletion continues, *gs* and transpiration decrease gradually. The generally more restricted stomatal control of conserving plants, leading to lower transpiration and assimilation rates, thereby results in lower productivity under well-watered conditions as compared with that of non-conserving types (Jones and Sutherland, 1991; Gallé et al., 2013; Moshelion et al., 2015).

The water-conserving (survivability-enhancing; Jones and Sutherland, 1991) strategy could be advantageous in barley growing regions with highly uncertain rainfall, whereas, in regions where rainfall is more predictable and less variable, the associated low transpiration and assimilation rate would produce distinctly lower yields than achievable by non-conserving plants (Galkin et al., 2018). One pillar of effective climate change adaptation is genetic improvement of crops and their cultivars targeted at the expected environmental conditions (He and Li, 2020; Peng et al., 2020; He et al., 2022). Breeding of improved, climate-resilient cultivars needs to keep pace with the changing environmental conditions (Rötter et al., 2015). Crop simulation models have the potential to provide the momentum that breeding needs to meet the demands for improved cultivars in a timely manner, because the models can create virtual genotypes and simulate their interaction with target environments and different management options, thereby assisting breeders in trait selection for yield improvement. To fully exploit their potential, crop models must accurately reproduce both physiological processes and their responses to a changing climate (Hammer et al., 2020; Peng et al., 2020; Ramirez-Villegas et al., 2020; Boote et al., 2021; Senapati et al., 2022). It is therefore necessary to increase our understanding of the physiological processes to evaluate and improve their representation in crop simulation models (Rötter et al., 2018; Peng et al., 2020).

This study was conducted to examine the water-response behavior and agronomic performance of four high-yielding European spring barley cultivars under intermediate drought. The selected cultivars were *a priori* assumed to show varying degrees of water-conserving and non-conserving behavior, based on previous trials. A recent study (Paul et al., 2023) has investigated the response of a different set of barley cultivars to two consecutive drought phases with a main focus on gene network analysis to understand the correlation of transcriptional and physiological drought responses in barley. Our study focuses on the effect of physiological drought responses on agronomic performance.

## 2 Materials and methods

### 2.1 Plant material and experimental setup

We selected three contrasting two-row spring barley cultivars intended for malting: cv. Chanell (CHAN), cv. Baronesse (BAR), and cv. Formula (FORM). Cv. RGT Planet (RGT) was added to the selection as one of Europe’s most popular cultivars (ragt.uk, 2022). The experiment started on 28 October 2022, with the sowing of seeds into small petri dishes (ø 100 mm). Seedlings were then transplanted to small seedling pots (50 mL). Two weeks later, at the two-leaf stage, they were transferred into 3-L pots (one seedling per pot). We used 1,250 g of potting mix [“Ökohum” containing plant compost, peat, and perlite; Organic matter = 80%, 160 mg/L N, 120 mg/L P_2_O_5_, and 320 mg/L K_2_0, pH = 5.8]. The pot capacity, i.e., the soil water-holding capacity after free drainage, was about 70%. For the early vegetative growth, all pots were randomly placed in climate chambers (PGC-105 CLF Plant Climatics GmbH, 1.5-m² area and 137-cm height) to ensure consistent growth conditions (14 h of light; temperature, 22.8°C/16°C). Watering was done *ad libitum*. Further crop protection and fertilization measures are shown in **Supplementary Table 1**.

On 9 December 2022, the pots were transferred to a plant chamber in a semi-controlled greenhouse at the University of Goettingen. During the whole experiment, artificial light (400-watt MT400DL/BH) was provided from 5:30 to 18:00 and the temperature was maintained at 23°C/18°C. The VPD fluctuated between 0.8 kPa and 2.4 kPa (mean, 1.7 kPa). On 27 January 2023, at heading (growth stage 50; Zadoks et al., 1974), all healthy plants with similar growth vigor and more than 12 tillers were placed on the Plantarray (Plant-Ditech, Yavne, Israel; **Supplementary Figure 1**) with two weather stations being installed at both sides (Atmos 14; Watchdog 2475) to monitor temperature, light, relative humidity, and VPD.

**Figure 1:**
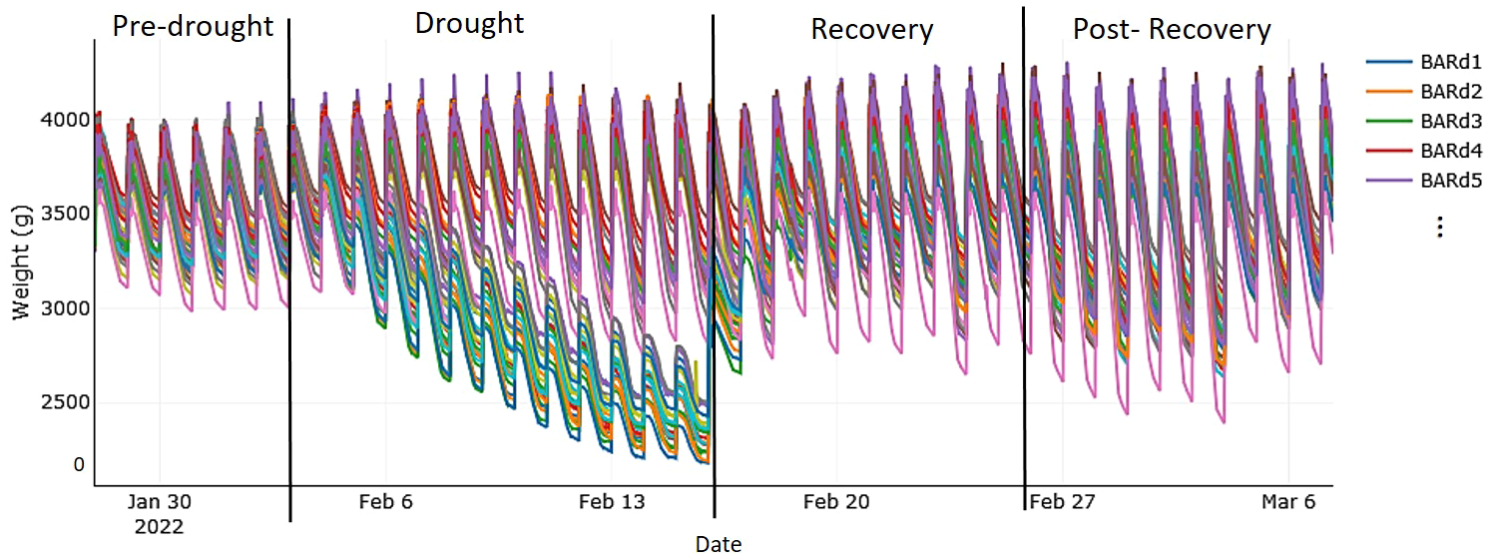
Autonomous, simultaneous, and continuous measurements of the system weight (i.e., weight of all components: plant, soil, pot, and all sensors) of each measurement unit taken from the first to the last day of the experiment running on the Plantarray (Plant-Ditech) and visualized with SPAC Analytics. The phases of the experiment were as follows: “pre-drought,” with well-watered (control) treatment (soil water content of each pot maintained at pot capacity) for 7 days; “drought,” with gradual deficit irrigation (80% of the plant's own previous day transpiration) for 12 days; “recovery period,” with control treatment for 10 days; and “post-recovery,” with slightly reduced irrigation to account for declining water demand of maturing plants. Each colored line represents one plant; as an example, the first five plants of drought-stressed cv. Baronesse are shown in the legend.

The Plantarray is a high-throughput functional phenotyping platform that continuously and simultaneously measures water flux in the soil–plant–atmosphere continuum. The system consists of individual, highly sensitive balances, each connected to their own control unit. As each measurement unit is connected to the water and fertilizer tank separately, individual irrigation and fertilization regimes are possible. Every 3 min, the weight of the whole system (i.e., pot, plant, and sensors; **Figure 1**, **Supplementary Figure 1**) is recorded and through internal calculations plant net weights, and a set of additional physiological plant parameters [e.g., daily transpiration (dTR), transpiration rate, and volumetric SWC] is provided. The data are made accessible in real time via the online analysis tool (SPAC Analytics), which can also be used for data visualization and analysis. The installed feedback irrigation system allows the user to establish a standardized drought treatment allowing for comparisons between the plants. Exposing all plants to similar drought stress is possible by taking into account each plant’s transpiration rate, e.g., by re-irrigating only a certain percentage of the previous day transpiration. This mimics the gradual development of soil water deficits in the field (Negin and Moshelion, 2016; Gosa et al., 2019; Dalal et al., 2020). To ensure that occurring water loss was solely due to plant transpiration, we covered the soil with a styrofoam sheet to prevent soil evaporation. A more detailed description of the system and the underlying theory can be found in Halperin et al. (2017) and Dalal et al. (2020).

### 2.2 Drought implementation and yield measurements

The plants were on the Plantarray for a total of 39 days. In the pre-drought phase (7 days; **Figure 1**), all plants were well watered (control treatment) by keeping the soil moisture of all pots at pot capacity through nocturnal irrigation cycles. Then, drought was implemented, at mid to end of heading (growth stage 55-59; Zadoks et al., 1974), by gradually reducing the daily irrigation amount to only 80% of the plants’ own previous day transpiration level (see gradual decline in volumetric SWC in **Supplementary Figure 2**). After 12 days, when all the plants had been exposed to similar soil–atmosphere stress conditions, we initiated the recovery phase in which irrigation followed the well-watered regime again. After 10 days of recovery, we slightly reduced irrigation to account for the declining water demand of the maturing plants. After 10 more days on the Plantarray (post-recovery phase), the plants were taken off and maintained in the greenhouse for maturation.

Upon harvest, on 20 April 2023, grain yield, biomass, thousand kernel weight (TKW), and seeds per spike were measured, and the harvest index (HI) was calculated. To determine dry weight, the plant material was dried at 80°C for 48 h, except for the grains, which were air-dried in paper bags to sustain fertility for future sowing.

### 2.3 Statistical analysis

The study was designed as a completely randomized experiment with four replicates for the well-watered (control) plants and five for the drought-stressed plants. The statistical analyses were run in R (version 4.2.1, R Core Team, 2021) and SPAC Analytics.

We checked the data for homoscedasticity using Levene’s test, the variance ratio test, and visual tools; to verify normality, we employed the Shapiro–Wilk test and visual tools (Zuur et al., 2009). Because the data of all parameters presented here were heteroscedastic but followed a normal distribution, we used the generalized least squares method to build the regression models. The only exception occurred for relative transpiration (rTR) during drought. Here, the violation of normality and homoscedasticity required a beta regression (Cribari-Neto and Zeileis, 2010). For all analyses, the significance level was set to p < 0.05. We conducted pairwise comparisons with the Tukey’s HSD (honestly significant difference) test using the R “emmeans” package (Lenth, 2023).

rTR was calculated by normalizing the measured daily sum of transpiration of the drought-stressed plants to the mean of the well-watered plants (per cultivar). For an assessment of drought resilience, we calculated each plant’s recovery rate as the slope of the linear regression between rTR and time [days of the recovery period; according to Galkin et al. (2018)]. Calculating the rTR allowed us to draw comparisons between the cultivars while accounting for possible differences in plant size, given that large plants would transpire more and deplete soil moisture faster than small plants and hence experience severe drought stress earlier (Galkin et al., 2018).

**Figure 2:**
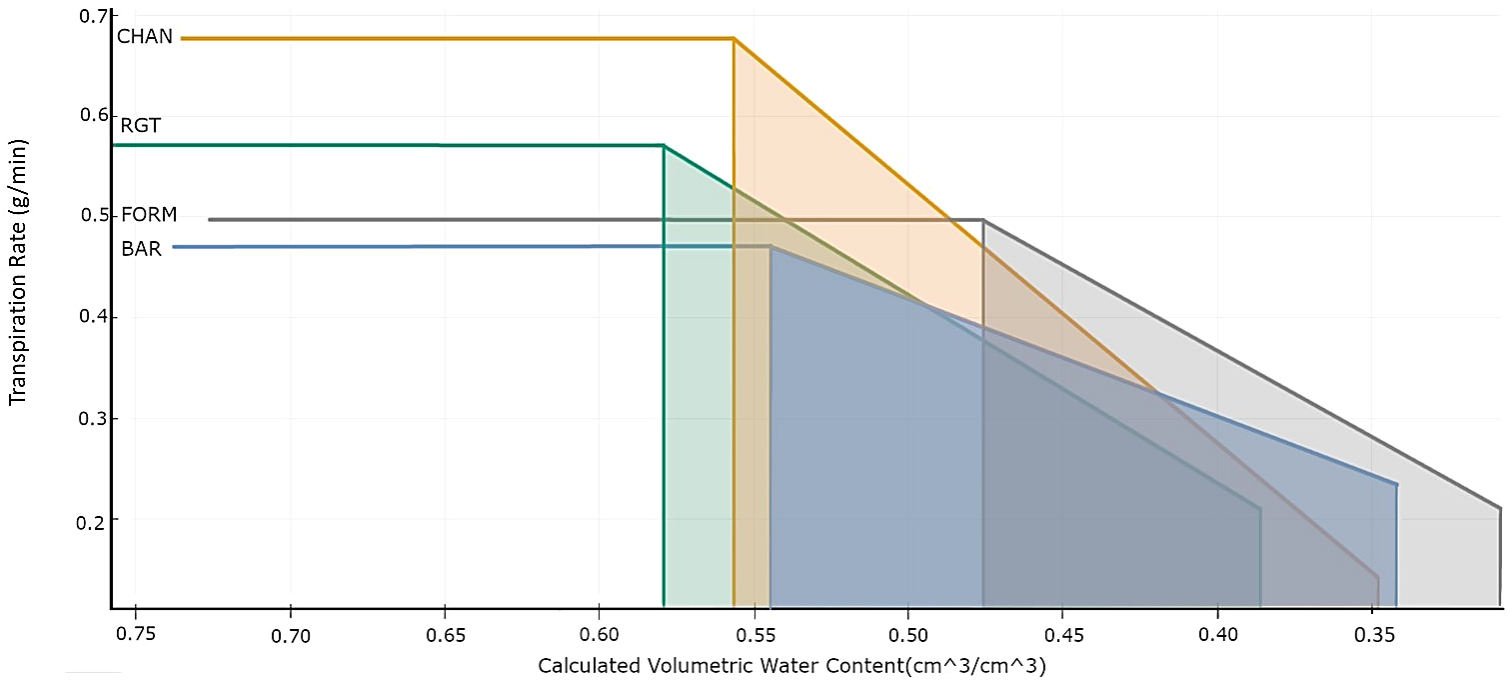
Bilinear model (R_2_: BAR = 0.5, CHAN = 0.94, FORM =0.75, RGT = 0.83) describing the relationship between transpiration rate (TR_rate, g/min) and volumetric soil water content (cm^3^/cm^3^) per cultivar. This shows the behavior of the drought-stressed plants from the day of drought implementation by gradual deficit irrigation (80% of the plant’s own previous day transpiration) to the last day of drought, with the horizontal line indicating the maximum transpiration rate (TRmax), the left vertical line indicating the breakpoint (θ_crit_), the vertical line on the right the terminal drought point, and the slope indicating the TR_rate reduction.

Comparability in plant responses can also be improved by analyzing the transpiration rate (TR_rate in g/min) when ambient conditions are most stable, in our case, 15:00–17:00 h, in relation to the volumetric SWC (in cm^3^/cm^3^, calculated by SPAC Analytics). This was done by fitting a bilinear regression model with the built-in algorithm of SPAC Analytics, considering only data points of the drought-stressed plants from the first to the last day of the drought phase. The resulting model (**Figure 2**) shows each cultivar’s θ_crit_, i.e., the point at which SWC becomes the transpiration-limiting factor (Halperin et al., 2017; Fu et al., 2022), and the slope of the TR_rate decrease after SWC is below θ_crit_. We called the last point on the falling part of the bilinear model the “terminal drought point.” This point, defined by the maximum transpiration rate (TRmax; horizontal part of the model before the breaking point), θ_crit_, and the slope of the TR_rate reduction, indicates the minimum TR_rate and corresponding minimum SWC each cultivar reached at the end of drought, right before the initiation of the recovery period. The size of the trapezoid area defined by θ_crit_, the x-axis, the slope of the TR_rate reduction, and the terminal drought point were calculated with integrals to get an estimate of the amount of water that the plant had transpired after θ_crit_ was reached. We calculated the corresponding cumulative sum of transpiration by extracting the respective time points for θ_crit_ and the terminal drought point from SPAC Analytics.

We calculated the average amount of dTR as cumulative sum of transpiration per phase (g)/phase duration (days) to account for the different durations of the experimental phases. To compare plant behavior under well-watered conditions, we calculated the dTR on the basis of the entire Plantarray phase (39 days). dTR and rTR were analyzed separately for each experimental phase. The stomatal conductance recorded by SPAC Analytics is based on the whole-plant level; therefore, it is also referred to as canopy conductance (see **Supplementary Figure 3**). For the analysis, days with missing data records were excluded.

Agronomic water-use efficiency (WUE) was calculated as harvest product (g)/water used during Plantarray phase (g). One plant (RGT, drought treatment replicate 2) had to be excluded from the physiological analysis due to an irrigation error causing faulty data. As the yield data were in accordance with the other observations, the replicate was still included in the agronomic analysis. We calculated the Pearson's correlation coefficient for cumulative transpiration and final grain yield.

In the Results section, all relevant observed results are reported irrespective of their statistical significance. Statistically significant (p < 0.05) results are indicated with exact p-values given in parentheses, and statistically non-significant results are indicated with *ns*, where suitable p-values of “almost significant” results are indicated.

## 3 Results

### 3.1 Transpiration during drought and recovery

**Figure 3:**
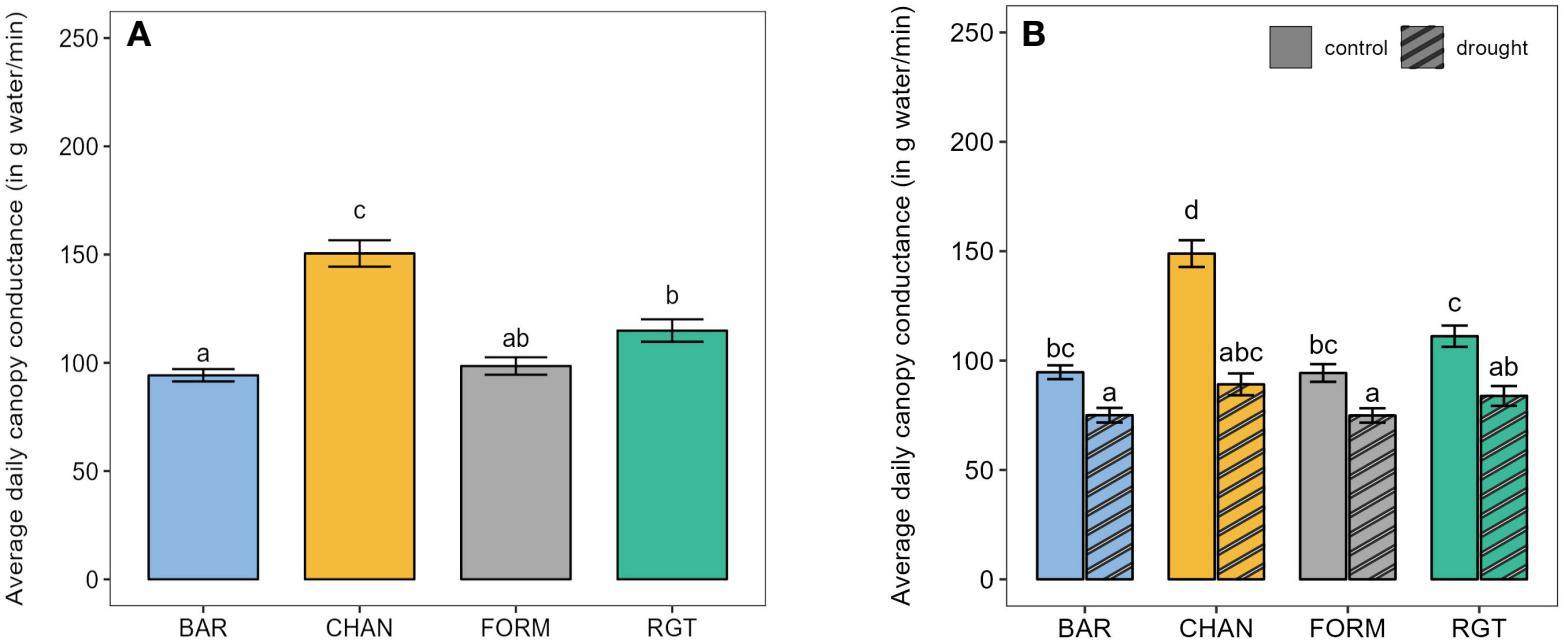
Canopy conductance. **(A)** Average daily canopy conductance of the well-watered control plants from the first to the last day on the Plantarray (39 days). **(B)** Average daily canopy conductance of well-watered control and drought-stressed plants during the 12-day drought phase. Letters indicate statistically significant differences between the groups (p < 0.05, pairwise comparisons Tukey’s HSD) and bars represent the standard error.

The highest canopy conductance under well-watered conditions (**Figure 3A**) was measured in CHAN (150.50 g/min, p < 0.001), followed by RGT (114.88 g/min), which showed about 15% higher values than FORM (*ns*) and BAR (p = 0.004). Drought reduced canopy conductance (p < 0.01) by 40.14% in CHAN, 24.57% in RGT, and around 21% in FORM and BAR (**Figure 3B**). Among the drought-stressed plants, canopy conductance in CHAN (89.14 g/min) was slightly higher (*ns*) than that in RGT (83.83 g/min); the lowest values were measured in FORM and BAR (≈ 75 g/min, *ns*).

**Figure 4:**
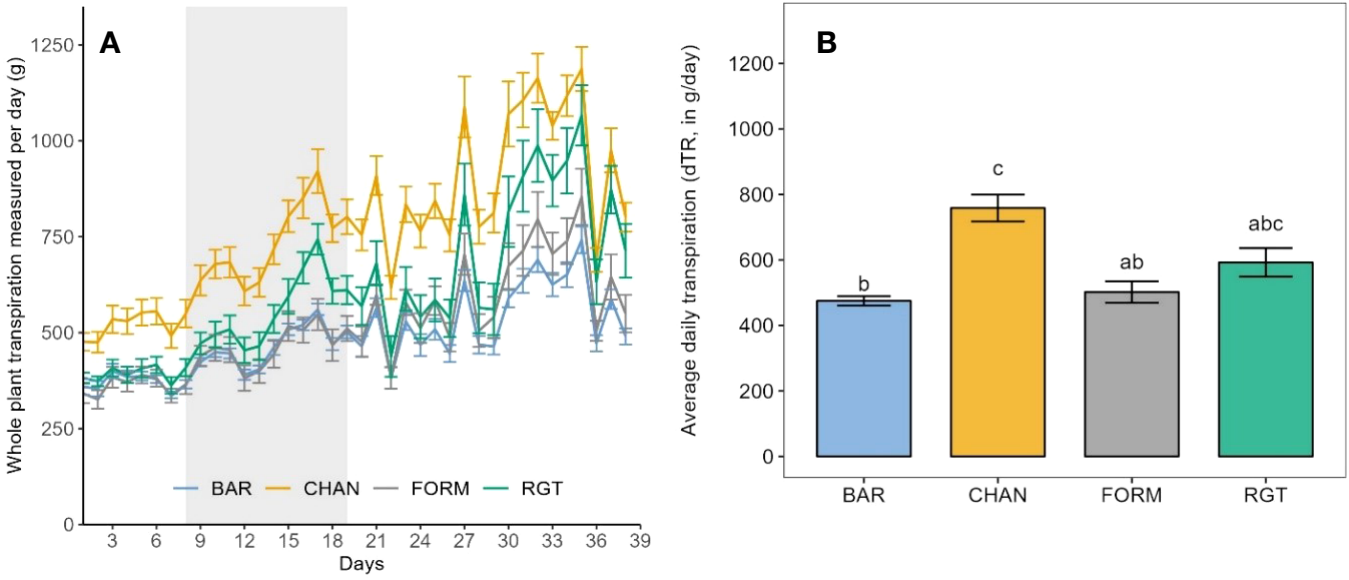
Transpiration of the well-watered control plants as observed during the whole experimental phase (39 days on the Plantarray). **(A)** Daily measurements of whole-plant transpiration from the first to the last day on the Plantarray. For comparison (with **Figure 5**) the 12-day drought period is also indicated (gray shaded area). **(B)** Average daily tanspiration. Letters indicate statistically significant differences between the groups (p < 0.05, pairwise comparisons Tukey’s HSD), and bars represent the standard error. The peaks in **(A)** are due to high PAR (photosynthetically active radiation) and VPD.

Under well-watered conditions, the highest dTR was observed in CHAN (758.431 g/day), which was more than 30% higher (p = 0.03) than the dTR measured in BAR and FROM. The second highest dTR (592.34 g/day, *ns*) was measured in RGT (**Figure 4**).

**Figure 5:**
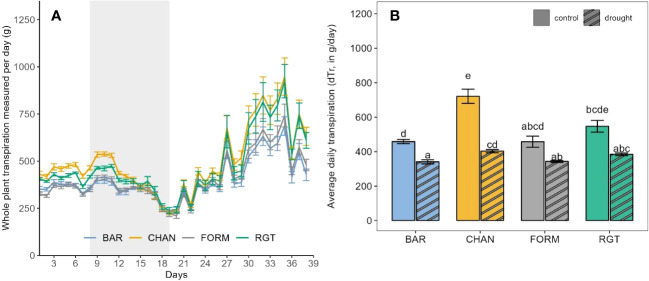
**(A)** Daily measurements of whole-plant transpiration of the drought-stressed plants from the first to the last day on the Plantarray (39 days) with the 12-day drought period implemented through gradual deficit irrgation (80% of the plant’s own previous day transpiration) indicated as gray-shaded area. **(B)** Average daily transpiration during the 12-day drought phase of drought-stressed vs well-watered control plants. Letters indicate statistically significant differences between the groups (p < 0.05, pairwise comparisons Tukey’s HSD), and bars represent the standard error. The behavior under well-watered conditions as observed during the whole experimental phase (on the Plantarray) is shown in **Figure 4**. The peaks in **(A)** are due to high PAR and VPD.

During the drought phase, notable reductions of dTR were observed in each cultivar (**Figure 5**). The strongest reduction occurred in CHAN (−44.15%, p = 0.02), followed by RGT (−29.81%, *ns)*, FORM (−25.0%, *ns*), and BAR (−25.65%, p = 0.003). The highest (*ns*) dTR among the drought-stressed plants was observed in CHAN (402.89 g/day) and RGT (384.05 g/day) and the lowest in FORM and BAR, both of which transpired about 15% (p < 0.04) less than CHAN. The lowest (p < 0.001) rTR during drought was measured in CHAN (0.58), whereas rTR of all other cultivars was around 0.75 (**Supplementary Figure 4A**).

**Figure 6:**
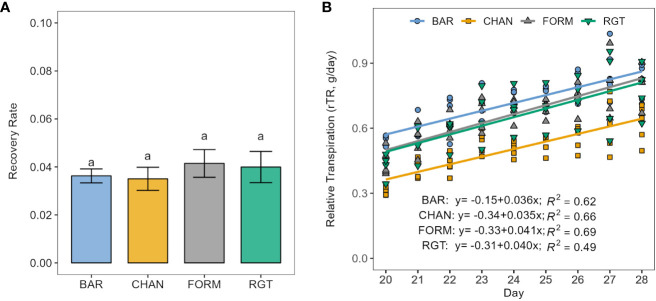
Recovery from drought stress (resilience) upon re-irrigation. **(A)** Recovery rate calculated as the slope of the linear regression between relative transpiration (rTR) and time in days of the recovery period. Letters indicate statistically significant differences between the groups (p < 0.05, pairwise comparisons Tukey’s HSD) and bars represent the standard error. **(B)** The respective linear models per cultivar.

FORM and RGT increased transpiration levels fastest (*ns*) upon re-irrigation (**Figure 6**), as indicated by the steepest slopes (0.041 and 0.040 rTR/d, respectively), whereas BAR (0.036 rTR/d) and CHAN recovered more slowly (0.035 rTR/d, *ns*). The rTR on the first day of recovery (day 20) was highest for BAR, followed by FORM, RGT, and, lastly, CHAN. This ranking remained until the last day of recovery.

During recovery, the lowest rTR was measured in CHAN (0.50, p < 0.001) and the highest in BAR (0.71, *ns*) followed by FORM and RGT (rTR ≈ 0.65, *ns*; **Supplementary Figure 4B**).

### 3.2 Transpiration rates in response to soil moisture depletion

The bilinear model (R^2^: BAR = 0.5, CHAN = 0.94, FORM = 0.75, and RGT = 0.83) fitted to describe the relationship between the TR_rate and the calculated SWC is shown in **Figure 2**. Under high soil moisture conditions, CHAN showed the highest TRmax (0.68 g/min), followed by RGT (0.57 g/min), FORM (0.50 g/min), and BAR (0.47 g/min). The highest θ_crit_ was observed for RGT (57.9%), followed by CHAN (55.7%), BAR (54.4%), and, lastly, FORM (47.6%). As soil moisture dropped below θ_crit_, the steepest decline in TR_rate was observed in CHAN (slope b = 2.56) and the most moderate one in BAR (b = 1.17). RGT (b = 1.71) and FORM (b = 1.87) were similar and ranked in between the other two cultivars. Moreover, FORM and BAR were still transpiring at very low soil moisture levels, whereas CHAN and RGT had already limited the TR_rate earlier (see terminal drought points in **Figure 2**). The shaded trapezoid area, indicative of the amount of water transpired after reaching θ_crit_, was largest for CHAN (0.08), followed by RGT (0.075) and BAR (0.071), while it was smallest for FORM (0.060). The corresponding cumulative sum of transpiration in the time period between θ_crit_ and the terminal drought point amounted to 4,205.24 g for CHAN, 4,117.28 g for RGT, 4,049.32 g for BAR, and 3,376.08 g for FORM. The lowest value of the terminal drought point was reached in CHAN, with a very low TR_rate (0.141 g/min) at SWC of 0.347 cm^3^/cm^3^. At almost the same SWC, the TR_rate of BAR (0.234 g/min) was notably higher. FORM’s TR_rate (0.211 g/min) was slightly lower than that of BAR at a comparatively lower SWC (0.308 cm^3^/cm^3^). The TR_rate of RGT (0.209 g/min) was close to FORM, yet the SWC was higher (0.385 cm^3^/cm^3^) at the terminal drought point.

### 3.3 Agronomic performance and WUE in response to drought

**Figure 7:**
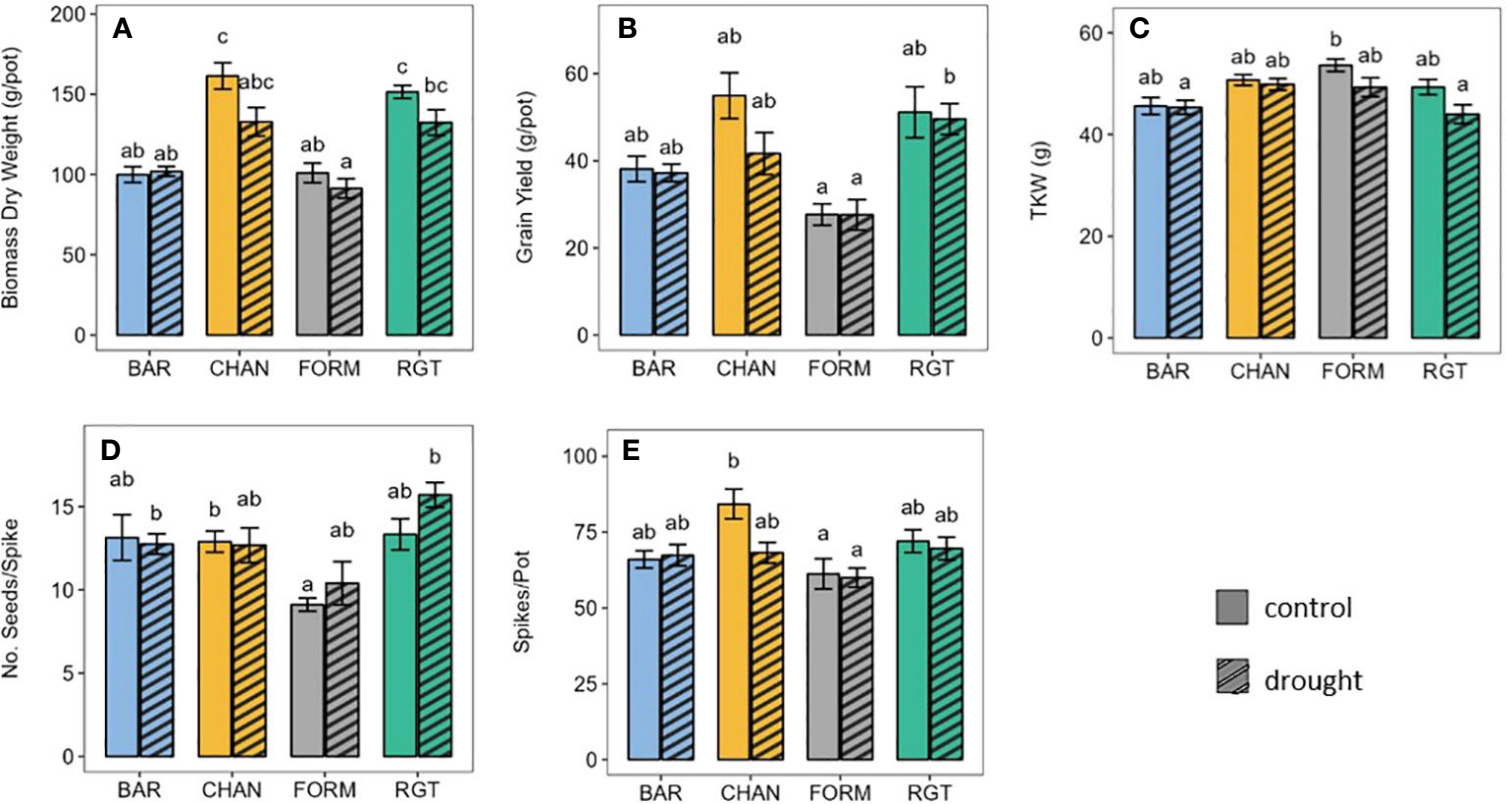
Agronomic parameters of well-watered (control) and drought-stressed plants. **(A)** Biomass, **(B)** grain yield, **(C)** thousand kernel weight, **(D)** number of seeds per spike, and **(E)** number of spikes per pot. Letters indicate statistically significant differences between the groups (p < 0.05, pairwise comparisons Tukey’s HSD), and bars represent the standard error.

Under well-watered conditions, CHAN (161.36 g per pot) and RGT (151.43 g per pot) produced more (p < 0.015) biomass than both FORM (101.02 g per pot) and BAR (99.91 g per pot; **Figure 7A**). The drought treatment altered (*ns*) biomass yield by −17.68% in CHAN, −9.59% in FORM, −8.90% in RGT, and +1.98% in BAR. Under drought, RGT (p = 0.045) and CHAN (p = 0.067, *ns*) produced about 30% more biomass than FORM and BAR (*ns*).

Under well-watered conditions, grain yield (**Figure 7B**) was highest (*ns*) for CHAN (54.96 g per pot), followed by RGT (51.15 g per pot), BAR (38.13 g per pot), and FORM (27.67 g per pot). Under drought, grain yield was reduced (*ns*) by 23.19% in CHAN, 2.38% in BAR, 0.39% in RGT, and 0.23% in FORM. Under drought (*ns*), the highest yields were thus obtained from RGT, lower yields were obtained from CHAN and BAR and the lowest yields from FORM. RGT yielded about 23.35% (p = 0.028) and CHAN about 14.06% (p = 0.063, *ns*) more than FORM. The yield of BAR deviated from CHAN by only 4.45 g per pot (*ns*).

The TKW (**Figure 7C**) under well-watered conditions (*ns*) was highest for FORM (53.6 g), followed by CHAN (50.7 g) and RGT (49.33 g), whereas the lowest TKW was measured in BAR (45.6 g). Under drought (*ns*), the ranking was similar, but the TKW was reduced by 0.57% in BAR, 1.66% in CHAN, 8.02% in FORM, and 11.51% in RGT.

Under well-watered conditions, the number of seeds per spike (**Figure 7D**) only deviated marginally (*ns*) between three of the cultivars (BAR and RGT = 13.13 seeds per spike and CHAN = 12.89 seeds per spike). The lowest seed number was produced by FORM under control (9.10 seeds per spike*, ns*) and under drought conditions. CHAN produced significantly more seeds per spike than FORM (p = 0.036) under well-watered conditions. Drought altered the number of seeds per spike (*ns*) by −2.93% in BAR and −1.66% in CHAN but +14.11% in FORM and +16.27% in RGT.

Under ample water supply, CHAN produced the most spikes per pot (**Figure 7E**), exceeding those of BAR (p = 0.057, *ns*) and FORM (p = 0.007) by 21 and 27% respectively and RGT by only 7%. Drought stress resulted in a reduction (*ns*) in the number of spikes per pot by 19% in CHAN. In all other cultivars, spike number only changed slightly ( ± 2%, *ns*).

**Figure 8:**
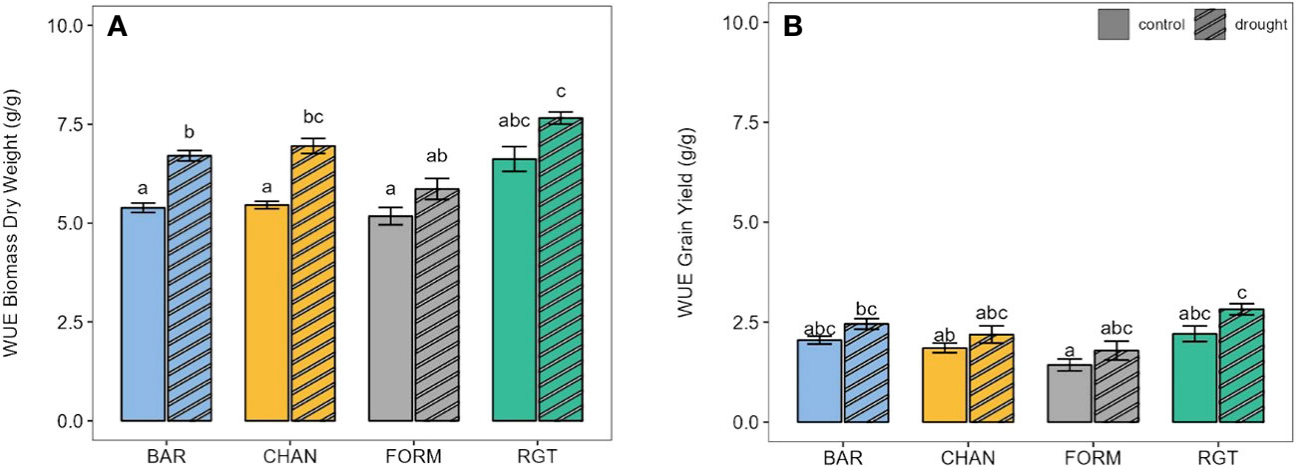
Water-use efficiency (WUE) of well-watered (control) and drought-stressed plants for **(A)** final biomass and **(B)** final grain yield. Letters indicate statistically significant differences between the groups (p < 0.05, pairwise comparisons, Tukey’s HSD) and bars represent the standard error.

In terms of WUE, biomass production under well-watered conditions (**Figure 8A**, *ns*) was most efficient in RGT (6.62 g/g), followed by CHAN (5.46 g/g), BAR (5.39 g/g), and, lastly, FORM (5.18 g/g). Under drought stress, WUE increased in all cultivars: by about 25% in BAR (p < 0.01) and CHAN (p < 0.01) and about 14% in FORM and RGT (*ns*). Under drought, the WUE of RGT exceeded that of BAR by 12% (p = 0.03), FORM by 23% (p = 0.01), and CHAN by 10% (*ns*). With ample water supply (*ns*), RGT (2.21 g/g) produced grains most efficiently (**Figure 8B**), followed by BAR (2.05 g/g), CHAN (1.85 g/g), and FORM (WUE, 1.43 g/g). Drought increased WUE (*ns*) by about 26% in FORM and RGT and about 19% in BAR and CHAN. WUE for grain yield under drought was highest in RGT (*ns*).

**Figure 9:**
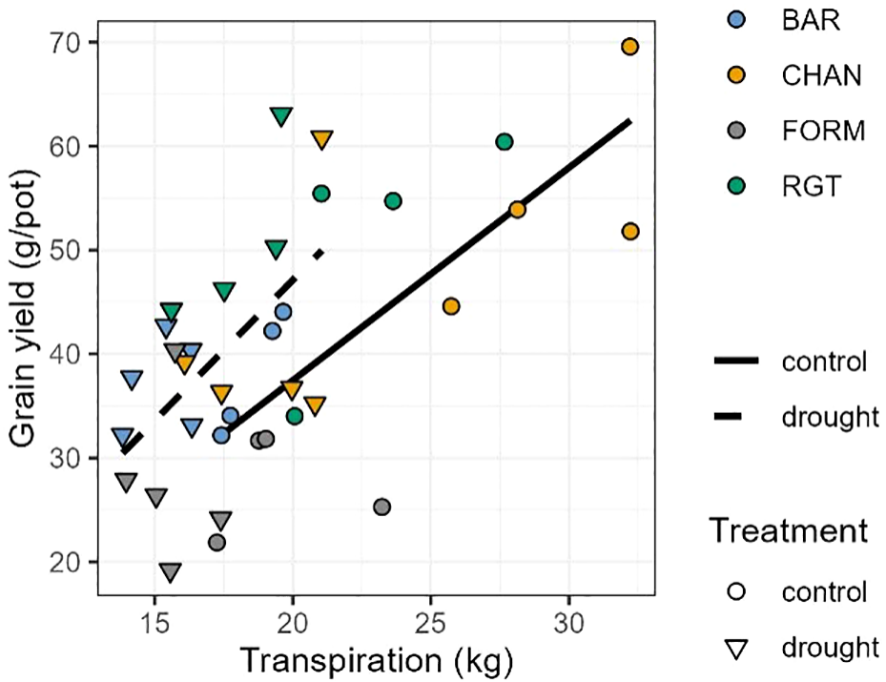
Pearson correlation between cumulative transpiration and grain yield. For well-watered (control; solid line and circles; r = 0.76, p < 0.01) and drought-stressed (dashed line and triangles; r = 0.55, p = 0.015) plants.

The positive correlation between cumulative transpiration and grain yield was consistent across treatments but was stronger under well-watered (control) conditions (control: r = 0.74, p = 0.0012) than under drought conditions (r = 0.54, p = 0.0177; **Figure 9**).

## 4 Discussion

Although water-conserving behavior might be associated with isohydric plant types and non-conserving with anisohydric types, drawing conclusions regarding the hydricity of our tested cultivars would only be possible if additional factors such as leaf water potential were taken into account (Sade et al., 2012; Moshelion et al., 2015). As our main focus was the agronomic performance, we prioritized comprehensive assessments of the whole plant to accurately describe its responsive behavior, rather than conducting destructive sampling for leaf water potential measurements. Here, we discuss all relevant (significant and non-significant) observed trends (for details regarding significance levels see Section 3).

### 4.1 Transpiration under drought

Under well-watered conditions, the stomatal conductance and transpiration level (see dTR and TRmax) is ordered, from high to low: CHAN > RGT > FORM > BAR. Physiological drought sets in when SWC starts to limit plant transpiration (θ_crit_; Halperin et al., 2017; Galkin et al., 2018). As SWC passed θ_crit_, CHAN reduced transpiration most rapidly, followed by FORM and RGT, which showed a similarly smooth transpiration decline, and, lastly, BAR. Overall, drought notably reduced canopy conductance in all cultivars (see **Figure 3**), which resulted in marked transpiration reductions, especially in CHAN (see dTR **Figure 5**). The strong transpiration decrease in CHAN points towards its non-conserving behavior, which entails stomatal closure near to the threshold for catastrophic xylem failure, as the strong reduction in canopy conductance under drought also indicates. Because water-conserving plants limit *gs* early on, a more gradual *gs* and transpiration decrease is sufficient. In our experiment, FORM showed water-conserving behavior and even more so BAR, which was the most conserving cultivar (Jones and Sutherland, 1991; Moshelion et al., 2015).

The most pronounced (and mostly significant) differences were found between non-conserving CHAN and the most conserving BAR (and, sometimes, FORM). RGT appeared to be an intermediate genotype, which, only in some cases, deviated notably (and significantly) from the two main water-use types discovered. Under well-watered conditions, with a transpiration level higher than FORM but lower than CHAN (see **Figures 3**, **4**; and TRmax, **Figure 2**), RGT displayed a less extreme variant of non-conserving water-use behavior. Interestingly, after the onset of drought, it did not reduce transpiration rapidly (like CHAN), but reduced it similarly to (and even slightly more gradually than) conserving FORM. As RGT switched from a rather non-conserving behavior under ample water supply to a more conserving behavior under drought, we consider it a dynamic water-use type. Such a dynamic (flexible) behavior has been observed in other species, e.g., grapevines (Zhang et al., 2012); however, the exact mechanisms facilitating this switch and corresponding thresholds are so far not understood. The dynamic behavior is worthy of further investigation, as it could be an ideotype trait for drought-prone environments (see Section 4.4; Sade and Moshelion, 2014; Moshelion et al., 2015).

The first cultivar to reach θ_crit_ was dynamic RGT, followed by non-conserving CHAN, most- conserving BAR, and, lastly, conserving FORM. Between plants with similar shoot traits and similar transpiration levels, differing root characteristics determine when θ_crit_ is reached (Gosa et al., 2019). Beneficial root architecture or superior biochemical and physiological processes facilitate greater soil water extraction and allow the plants to delay the point of physiological drought (Lynch et al., 2014; Gosa et al., 2019).

In our experiment (**Figures 2**, **7A**), the biomass of CHAN was similar to RGT, yet its transpiration was considerably higher; consequently, one would expect CHAN to reach θ_crit_ at higher SWC than RGT. However, in fact, the opposite was the case, which suggests that CHAN might have more favorable root traits than RGT (same for FORM vs. BAR), a question that has to be explored in further experiments. The θ_crit_ values of most conserving BAR and non-conserving CHAN were quite similar, yet, at a SWC of 35%, CHAN had already reduced transpiration notably (**Figure 2**). The more gradual TR_rate reduction (combined with lower pre-drought TRmax) allowed BAR to maintain a comparatively higher transpiration at a low SWC, at which non-conserving CHAN was already close to desiccation (Sade et al., 2012; Moshelion et al., 2015). Dynamic RGT and conserving FORM decreased transpiration at a similar rate, resulting in similar transpiration levels at the terminal drought point. However, FORM’s θ_crit_ occurred at much lower SWC, which delayed the decline of transpiration and extended FORM’s transpiration period. Whether such extended periods of transpiration rendering a physiological benefit ultimately translate into agronomic advantages depends on many factors, as discussed below.

### 4.2 Drought recovery

Drought resilience can be defined as the plant’s ability to recover from drought stress by resuming growth, including re-initiation of transpiration and photosynthetic processes after re-watering. The degree to which a plant can recover depends *inter alia* on the plant’s resistance to xylem cavitation, to damages to the photosynthetic system (Johnson et al., 2018; Qi et al., 2021), to damaging ROS (reactive oxygen species) levels (Cruz de Carvalho, 2008), and to leaf and root loss, as well as on the duration and severity of the stress (Sade et al., 2012; Moshelion et al., 2015; Johnson et al., 2018; Qi et al., 2021). Despite recovering at almost the same slow rate as non-conserving CHAN, conserving BAR could recover better overall [see high rTR at the end of the recovery phase (**Figure 6B**) and higher rTR during recovery (**Supplementary Figure 4B**)], one reason being that it entered the recovery period with a higher transpiration level (see TR_rate at terminal drought point in **Figure 2**; see also **Figure 6B**). In contrast, CHAN recovered poorly, perhaps because of xylem damage (Johnson et al., 2018). Conserving FORM and dynamic RGT showed a slightly faster recovery rate than BAR, yet, presumably due to their lower transpiration at the terminal drought point, they could not recover to the same extent as BAR (**Figure 6B**; **Supplementary Figures 4B**).

Previous studies have associated conserving cultivars with better recovery as compared with non-conserving ones (Galkin et al., 2018; Dalal et al., 2019), which is confirmed in our study. The two conserving cultivars and the dynamic one, which follows a “conserver-like” transpiration reduction after the onset of physiological drought, recovered better (see rTR **Supplementary Figure 4B**) and faster (see slope of rTR increase **Figure 6**) than the non-conserving cultivar. Further research should investigate the question of whether delayed xylem failure, or another factor, such as greater resistance to drought induced damage (Johnson et al., 2018; Qi et al., 2021) or upregulation of certain aquaporins (Patel and Mishra, 2021), accounts for higher transpiration levels at the terminal drought point (as observed in the conserving cultivars) and faster or better recovery.

### 4.3 Yield and WUE

**Figure 10:**
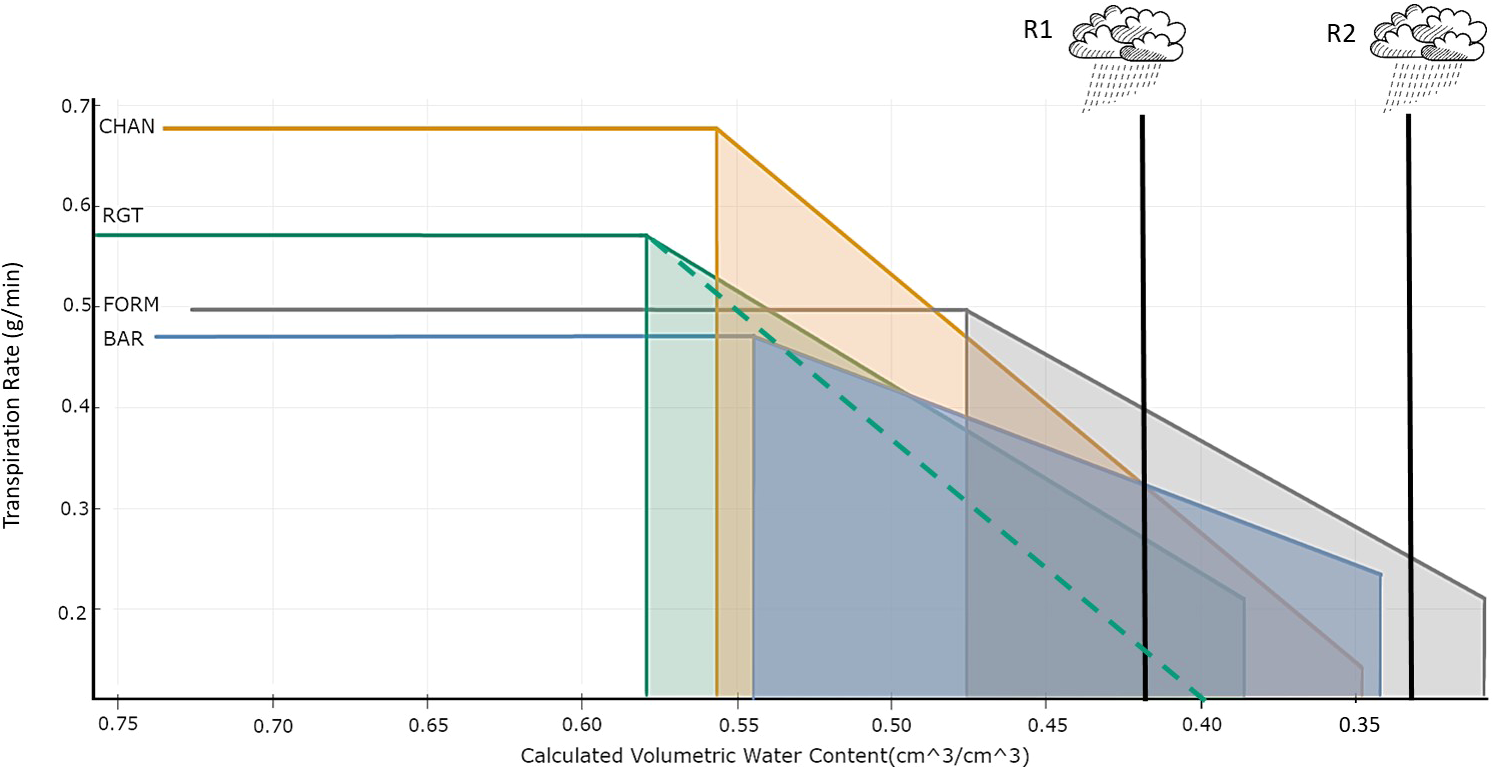
Risk management in an uncertain environment. Bilinear model describing the relationship between transpiration rate (TR_rate, g/min) and volumetric soil water content (cm^3^/cm^3^) per cultivar. The horizontal line indicates the maximum transpiration rate (TRmax). The breakpoint (left vertical line) indicates θ_crit_. The vertical line on the right indicates the terminal drought point. The slope shows the reduction of the TR_rate. Two hypothetical rain events (R1 and R2) are shown. The dotted green line indicates the hypothetical TR_rate decrease of RGT had it followed the same non-conserving behavior as CHAN and decreased TR_rate with the same steepness.

Although only a few results of the agronomic analysis were statistically significant, the observed trends aligned well with literature. Under well-watered conditions, the grain yield, from high to low, spanned non-conserving CHAN > dynamic RGT > very conserving BAR > conserving FORM, following the well-established relationship whereby high *gs* facilitates high gas exchange rates and therefore high yield (**Figure 10**) (Howell, 1990; Kemanian et al., 2005; Moshelion et al., 2015). We assume that the main driver for the overall poor yield in FORM was probably the lower grain number per spike, which could not be compensated for by the higher TKW (Kennedy et al., 2017).

High yield stability is important to ensure high cereal productivity under changing climate conditions (Powell et al., 2012; Moualeu-Ngangué et al., 2020). In non-conserving CHAN, the drought stress conditions generated losses in biomass, spike number, and, ultimately, grain yield. Plants that produce a lot of biomass, like CHAN, aim to reduce the transpiring leaf area under drought stress, e.g., by shedding or reducing the size of leaves (Chaves et al., 2003; Blum, 2005; Varga et al., 2017), which strongly affects grain yield through a reduction in light interception (Araus et al., 2002; Farooq et al., 2009; Abid et al., 2018). The comparatively higher biomass of CHAN possibly also resulted in greater nighttime respiration, which might have contributed to its high yield loss (Sadok and Tamang, 2019; López et al., 2021). However, due to the nocturnal irrigation cycles in our experiment, nighttime respiration could not be measured as it would not have been possible to differentiate between weight increments due to irrigation and weight loss due to plant respiration or soil drainage. This will be the subject of future experiments where irrigation schemes will be changed accordingly.

Marginal to no yield losses were observed in BAR, FORM, and RGT. This could have been due to their more gradual decline of *gs* and transpiration after the onset of drought, preventing permanent, yield-reducing damage (Weldearegay et al., 2016) or due to certain degrees of drought tolerance as observed by Pecio and Wach (2015). They reported on drought-tolerant barley cultivars in which enhanced productivity correlated with increased grain weight and reduced grain number. Possibly, an inverse compensation mechanism preventing larger yield losses (**Figures 7C, D**) took effect in FORM and RGT; this requires further investigation. Another possible reason could be that, for these three cultivars, the implemented drought stress was not severe enough to result in greater yield effects. However, further extending the drought period was not possible because CHAN had already shown severe signs of drought stress and the transpiration level of the drought-stressed plants was at 30% of the control on the last day of drought, which was the threshold set for re-irrigation [see, e.g., Paul et al. (2023)]. The other cultivars had not reached that threshold yet.

Possibly, the dynamic water-use behavior observed in RGT was the main reason for its highest (although *ns*) WUE (also see Section 4.4). CHAN showed a higher grain yield but with lower WUE than conserving BAR (*ns*), which was probably due to CHAN producing much more biomass and accordingly transpiring more (**Figures 5B**, **2**; Varga et al., 2017; Moualeu-Ngangué et al., 2020).

### 4.4 Implications for breeding

Under well-watered conditions and mild-to-moderate drought stress, non-conserving plants that follow a productivity-maximizing behavior outperform water-conserving plants striving for survivability, in terms of yield and plant growth, due to the high level of transpiration and net CO_2_ assimilation (Jones and Sutherland, 1991; Sade et al., 2012; Moshelion et al., 2015). Which of those two strategies results in more stable yields under severe or prolonged drought conditions depends, *inter alia*, on the level of soil dryness, the water-holding capacity of the soil, and the likelihood of the next rainfall event (Galkin et al., 2018; Ludwig and Asseng, 2010).

As an illustration, we discuss two hypothetical scenarios (**Figure 10**), with one rain event occurring when SWC is depleted to 42% (R1) and another (R2) when an extended drought has reduced SWC to 32%. From the onset of drought (θ_crit_) to the R1 event, non-conserving CHAN has a high transpiration and assimilation rate, which generates a “productivity head start” in comparison to the other cultivars. This will ultimately result in an overall agronomic advantage when rain occurring at R1 rehydrates the soil and allows plants to (fully or partially) recover to pre-drought assimilation rates (Sade et al., 2012). If, however, the drought period is extended and instead rain only occurs at R2, then non-conserving CHAN will already be close to desiccation, whereas conserving BAR will still be able to conduct gas exchange at comparatively high levels. This can render a significant advantage under prolonged drought.

Depending on the severity of the drought-induced damage (especially in CHAN) and the potential to recover to pre-drought assimilation levels after R2 (in CHAN and BAR), the agronomic advantage may belong to the conserver (BAR) rather than the non-conserver (CHAN) (Sade et al., 2012). Yield-reducing physiological damage could be minimal in the conserver, which may be able to return almost fully to pre-drought assimilation rates. The non-conserver, however, might have suffered severe damage and may only reach 50% of the pre-drought assimilation. In such a case, the conserver may ultimately produce more yield than the non-conserver, which possibly might have happened in our experiment for BAR and CHAN, if we had prolonged the drought period even further. These considerations illustrate that drought duration is a crucial factor for assessing the suitability of the different water-use types for specific environments. A favorable addition to a conservative water-use behavior is a beneficial root system, which delays the onset of physiological drought (θ_crit_) and allows the plant to extract water from increasingly dry soil (FORM; **Figure 10**). In an optimal case, a cultivar could avoid substantial transpiration reduction during drought and show fast and full recovery after the next rain (**Figure 10**; see Section 4.2; Moshelion et al., 2015; Negin and Moshelion, 2016).

We conclude that water-conserving strategies are more suitable for extended dry spells, e.g., during the pre-anthesis phase, if: (i) the precipitation during the subsequent reproductive stage is likely to be sufficient for unlimited growth; or (ii) if water saved during the pre-anthesis phase can be stored effectively by the soil to compensate for water deficits emerging during the reproductive stage (Sinclair, 2018). Non-conserving water use is suitable primarily for environments where only short dry spells prevail. A combination of both behaviors, as in a dynamic water-use type, could be ideal for combining high productivity and high drought resilience. Such a plant, resembling the behavior of RGT, would conserve water under drought and recover quickly, in the best case to pre-drought transpiration levels, after the next rain (Moshelion et al., 2015; Negin and Moshelion, 2016), and would be especially suited to environments with intermittent droughts. Within the cultivars examined in this study, we conclude that the dynamic water use of RGT played an important role for its high WUE and resilience. Its rather non-conserving water use before drought gave RGT a productive advantage over the more conserving cultivars, whereas responding like a water-conserver to drought made it more productive than non-conserving CHAN.

Because these conclusions are derived from a pot experiment conducted in a semi-controlled environment, they cannot be directly translated into breeding recommendations but rather need to be both corroborated through field studies (Passioura, 2006; Poorter et al., 2016) complemented by insights from modeling [see, e.g., Sinclair (2018) and Boote et al. (2021)] and also tested under more severe drought stress conditions than the ones imposed in our experiment. Nonetheless, our experiment provides valuable new insights into the drought-response behavior of spring barley and can guide prospective modeling studies, e.g., evaluating the currently implemented process descriptions of drought-response behavior.

## 5 Conclusions

We gained new insights into water-use behavior of European spring barley cultivars by examining their response to intermediate drought using a high-throughput functional phenotyping platform. By exhibiting a non-conserving behavior under ample water supply, the assimilate production of dynamic water users, such as RGT, exceeds that of water-conserving cultivars, which have a much lower transpiration rate. However, at the onset of drought, dynamic water users switch to a more water-conserving behavior by only gradually decreasing stomatal conductance, allowing them to maintain assimilation for longer periods of time and to be more productive than non-conservers. Combining a dynamic water-use behavior with better drought resilience traits and favorable root traits that delay the point at which the plant senses drought (the critical soil moisture level) could create an ideotype for intermediate droughts occurring at around flowering.

## Data availability statement

The raw data supporting the conclusions of this article will be made available by the authors, without undue reservation.

## Author contributions

IA, ED, AS, MM, and RR planned and designed the research. ED and IA performed the experiment and collected the data. MA prepared, analyzed, and visualized the data and wrote the manuscript. IA, AS, MM, AD-G, ED, GB-M, and RR reviewed and edited the manuscript. All authors contributed to the article and approved the submitted version.
