# Supplementary material for: Drought response of water-conserving and non-conserving spring barley cultivars
Source: Front Plant Sci. 2023 Oct 24;14:1247853. doi: 10.3389/fpls.2023.1247853 (PMC10628443; doi:10.3389/fpls.2023.1247853)
Supplement: Supplementary file 1 [file DataSheet_1.docx]

# Supplement

**Drought response of water-conserving and non-conserving spring barley cultivars**

**Mercy Appiah^1^, Issaka Abdulai^1^, Alan Schulman^2,3^, Menachem Moshelion^4^, Elvira S. Dewi^1,5^Agata Daszkowska‑Golec^6^, Gennady Bracho-Mujica^1^ , Reimund P. Rötter^1,7^**

*1- University of Göttingen, Department of Crop Sciences, Tropical Plant Production and Agricultural*

*Systems Modelling (TROPAGS), Grisebachstr. 6, 37077 Göttingen, Germany*

*2 – Natural Resources Institute Finland (Luke), Latokartanonkaari 9, FI-00790 Helsinki, Finland*

*3 - Institute of Biotechnology and Viikki Plant Science Centre, University of Helsinki, Yliopistonkatu 3, 00014, Finland*

*4 - Institute of Plant Sciences and Genetics in Agriculture, The Robert H. Smith Faculty of Agriculture, Food and Environment, The Hebrew University of Jerusalem, Rehovot 76100, Israel;*

*5-Universitas Malikussaleh, Department of Agroecotechnology, Faculty of Agriculture, 24355 Aceh Utara Indonesia,*

*6- Institute of Biology, Biotechnology and Environmental Protection, University of Silesia in Katowice,
Jagiellońska 28, 40-032 Katowice, Poland*

*7 University of Göttingen, Centre for Biodiversity and Sustainable Land Use (CBL), Büsgenweg 1, 37077 Göttingen, Germany*

Table. 1 Crop protection and fertilization measures taken during the vegetative growth period before the plants were transferred to the Plantarray at the heading stage.

| **Date** | **Type/Amount** |
| --- | --- |
| 19 Nov 2022 | NPK fertilizer Hakaphos Blau 15-10-15; concentration: 8g fertilizer/l water; amount: 50 ml/pot |
| 06 Jan 2023 | NPK fertilizer Hakaphos Blau 15-10-15; concentration: 10g fertilizer/l water; amount: 100 ml/pot) |
| 20 Jan 2023 | NPK fertilizer Hakaphos Blau 15-10-15; concentration: 12g fertilizer/l water; amount: 250 ml/pot |
| 27 Jan 2023 | NPK 8- 8- 6, Wuxal Super; concentration (0.2ml / l water ) , fertilization with irrigation on the plant array system |
| 18 Feb 2023 | Spraying against aphids Teppik 0.5 g/1 L of water |


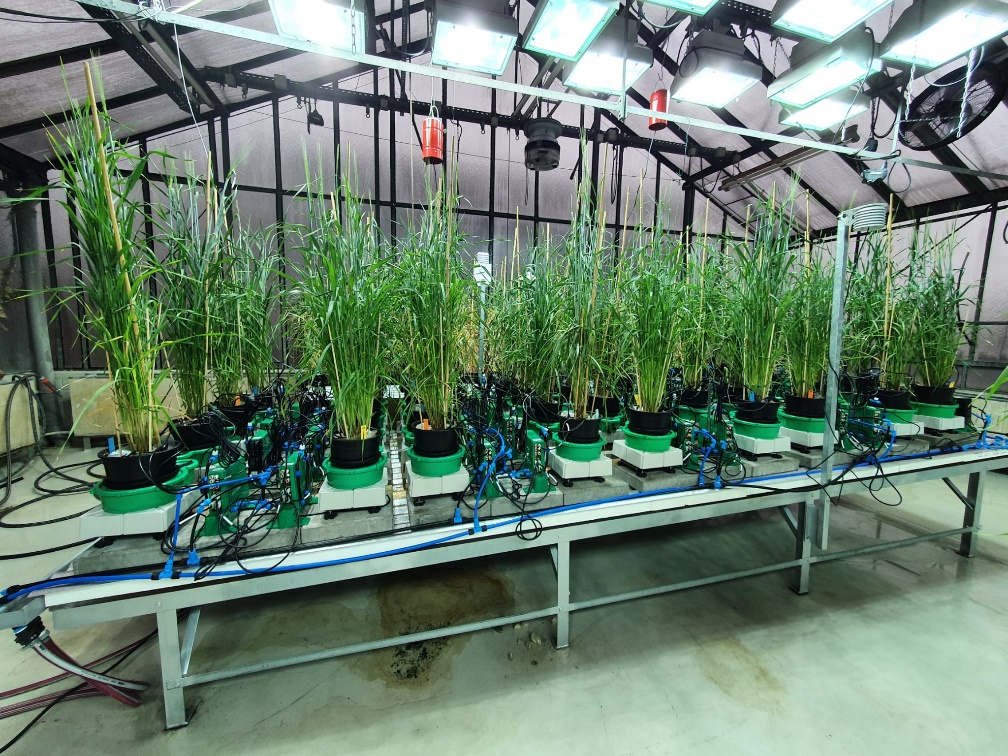


SFig. 1 The four spring barley cultivars on the Plantarray system in the semi-controlled greenhouse of TROPAGS Institute at the University of Goettingen in 2022


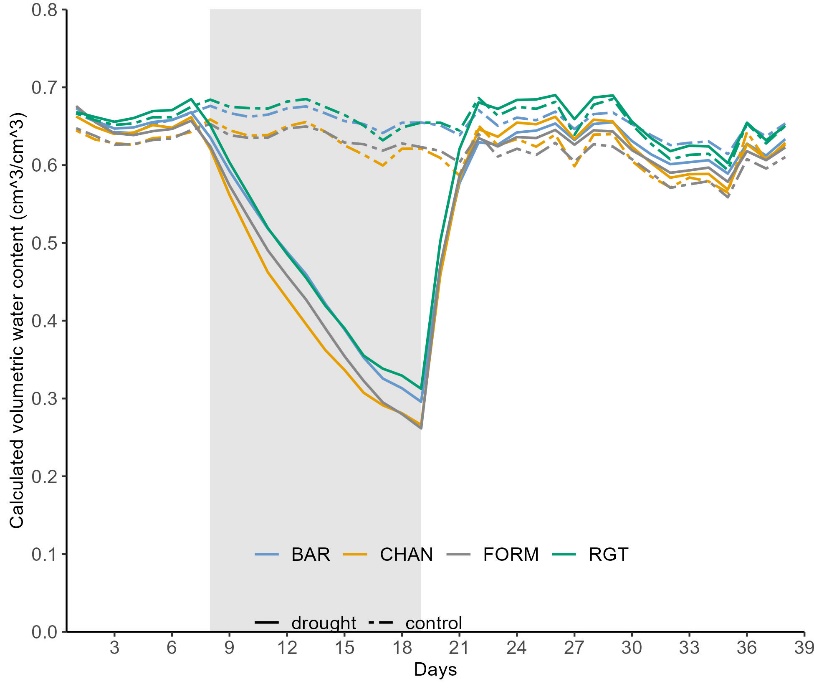


SFig. 2: Volumetric soil water content of control plants (solid line) and drought stressed plant (dotted line) during the 7 day pre-drought phase, the 12 day drought phase (grey shaded area), recovery (10 days ) and post-recovery (10 days).


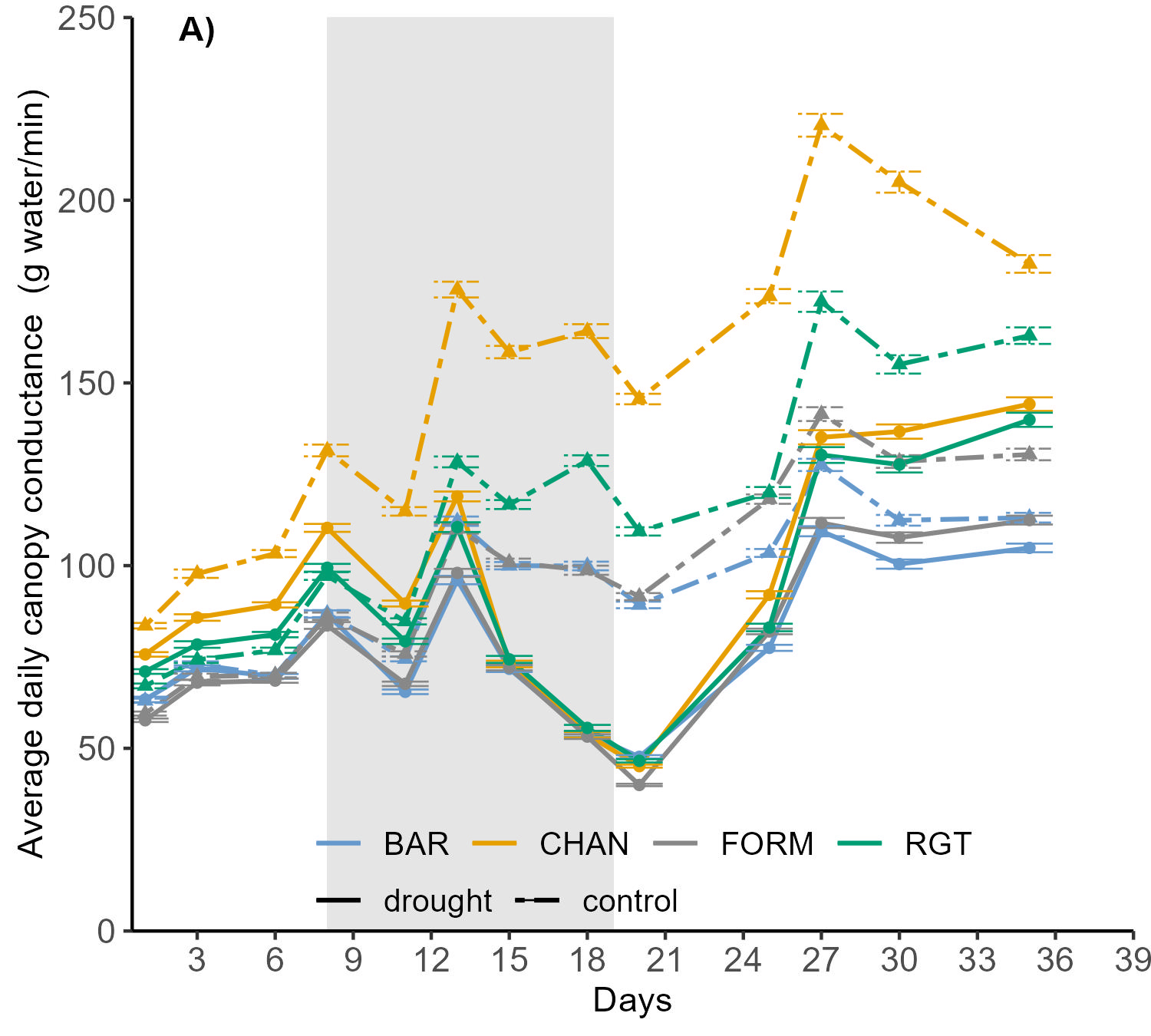


SFig.3 Daily canopy conductance from the first to the last day on the Plantarray (39 days) with a 12 day drought period implemented through gradual deficit irrigation (80% of the plant’s previous day transpiration) indicated as grey shaded area; Control plants (dotted line ) and drought stressed plants (solid line) are shown and the standard errors of the mean are indicated with error bars. The peaks in daily canopy conductance are due to high PAR and VPD.


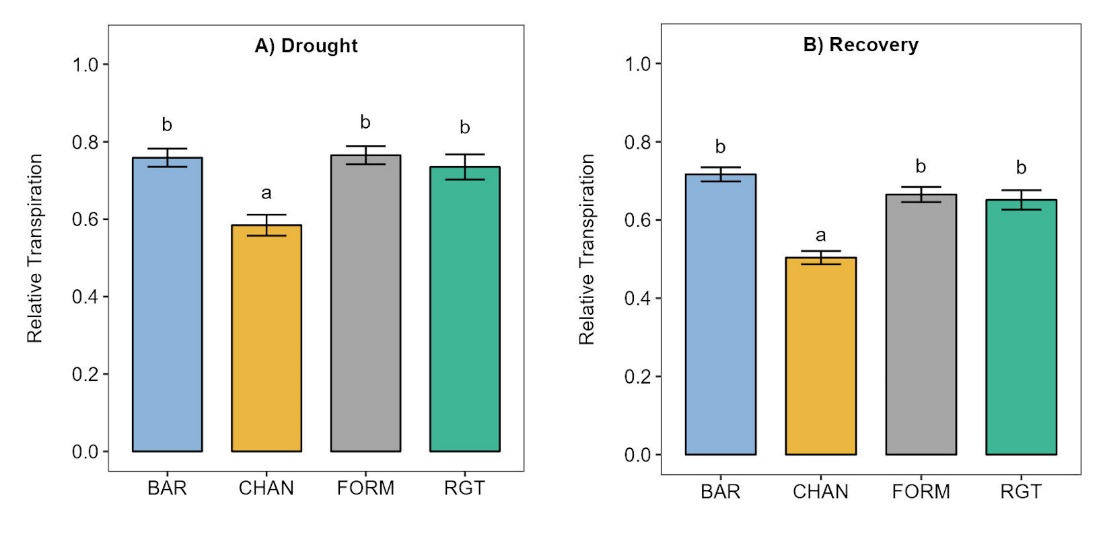

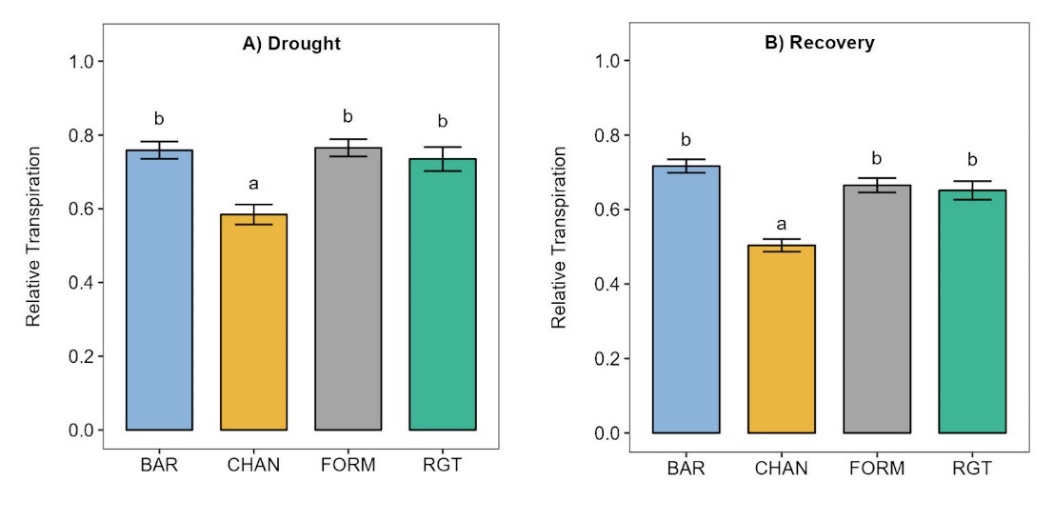


SFig.4 Relative transpiration (rTR, calculated as the transpiration of the drought stressed plants normalized to the mean transpiration of the well–watered plants) rTR during A) drought and B) recovery phase. Letters indicate statistically significant differences between the groups (p<0.05, pairwise comparisons Tukey’s HSD) and bars represent the standard error.
